# Research trends and hotspots of exosomes in respiratory diseases

**DOI:** 10.1097/MD.0000000000035381

**Published:** 2023-09-29

**Authors:** Jinjie Fu, Wenjie Song, Zheng Hao, Mengzhen Fan, Yang Li

**Affiliations:** a Graduate School, Tianjin University of Traditional Chinese Medicine, Tianjin, China; b College of Traditional Chinese Medicine, Tianjin University of Traditional Chinese Medicine, Tianjin, China; c Medical History and Literature Center, Tianjin University of Traditional Chinese Medicine, Tianjin, China; d Tianjin Key Laboratory of Modern Chinese Medicine Theory Innovation and Transformation, Tianjin, China.

**Keywords:** bibliometrics, exosomal miRNA, exosomes, lung cancer, mesenchymal stem cells, respiratory disease

## Abstract

Currently, theoretical studies on exosomes in respiratory diseases have received much attention from many scholars and have made remarkable progress, which has inestimable value and potential in future clinical and scientific research. Unfortunately, no scholar has yet addressed this field’s bibliometric analysis and summary. We aim to comprehensively and profoundly study and explore the present situation and highlights of exosome research at the stage of respiratory diseases and to provide meaningful insights for the future development of this field. The WOSCC literature was gathered for the study using bibliometrics, and the data were collected and analyzed using CiteSpace, VOSviewer, Microsoft Excel, and Endnote software. The publication language is “English,” and the search strategy is TS = (exosome OR exosomes OR exosomal) AND TS = (respiratory OR lung). The search time is from the beginning of the WOS construction, and the deadline is July 11, 2022, at 22:00 hours. The literature types selected were dissertation, review paper, and online published paper. The analysis includes 2456 publications in 738 journals from 76 countries, 2716 institutions, and 14,568 authors. The field’s annual publications have been rising, especially in recent years. China and the US lead research, and prominent universities, including Harvard Medical School, Shanghai Jiao Tong University, and Fudan University, are essential research institutes. Takahiro Ochiya, whose research focuses on exosomes and lung cancer, and Clotilde Théry, a pioneering exosome researcher, are the most cited authors in this field. The key terms include lung cancer, non-small cell lung cancer, mesenchymal stem cells, intercellular communication, exosomal miRNAs, and oncology. Cell biology, biochemistry & biotechnology, and oncology are related fields. The final summary of research hotspots is exosomes and lung cancer, mesenchymal stem cell-derived exosomes and lung inflammation, and miRNAs in exosomes as biomarkers for respiratory illnesses. The present research situation and relevant hotspots of the area were analyzed through bibliometric studies on exosomes in respiratory diseases. The research development in this field has a considerable upside, and the exosome’s function in diagnosing, treating, monitoring, and prognosis of respiratory illnesses cannot be taken lightly. Moreover, we believe the research results will bring the gospel to many patients with clinical respiratory diseases shortly.

## 1. Introduction

Respiratory diseases have long been the cause of millions of deaths worldwide, with some scholars showing in The Lancet Respiratory Medicine that the number of people suffering from chronic respiratory diseases was as high as 545 million worldwide in 2017, up 39.8% compared to 1990.^[1]^ Nowadays, despite our deepening research on the underlying mechanisms leading to the pathogenesis of lung diseases and advances in new methods, such as nanomedicine and regenerative medicine, which have helped to diagnose or treat various lung diseases,^[2–5]^ there are still many shortcomings in the detection, control, and prognosis of some respiratory diseases. Most treatments are still limited to symptomatic treatment and limiting disease progression.

However, the continuous progress of exosome research results has given many scholars new insights into the pathogenesis, diagnosis, and treatment of respiratory illnesses. First, exosomes play a vital role in the pathogenesis of respiratory diseases: In recent years, some scholars have found that exosomal miRNAs and lncRNAs play a non-negligible role in the pathogenesis of various respiratory diseases such as lung cancer, chronic obstructive pulmonary disease (COPD), asthma, tuberculosis, and interstitial lung disease.^[6–8]^ For example, in asthma studies, it was found that exosomes appear to activate the immune response in asthmatics,^[9]^ while in COPD, some researchers found that miRNAs can be transferred by exosomes, which in turn induce disease in target cells.^[10]^ Regarding idiopathic pulmonary interstitial fibrosis, although the pathogenesis of exosomes and the disease has not been fully described, it has been found through the establishment of in vitro and in vivo mouse model tests that exosomes may intervene in the development of the disease.^[11]^ Second, exosomes are an innovative identification, and diagnostic tool for respiratory diseases: exosomal miRNAs and lncRNAs can express different patterns under different conditions of physiology and pathology, which may suggest that exosomes possess the capacity to reflect illnesses conditions.^[12]^ Some researchers have even suggested that exosomal miRNAs possess a chance to serve as biomarkers and targets of therapy for noninvasive diagnosis of respiratory diseases in the future due to their finding that the profiles of exosomal miRNAs in patients with lung illnesses are significantly different from those in healthy individuals.^[13–15]^ Other scholars compared the number of exosomes in bronchoalveolar lavage fluid (BALF) in patients with lung nodules and normal healthy humans by setting up a test group. They found that the number of exosomes in BALF in patients with lung nodular disease was significantly higher than that in healthy individuals, indicating that the disease affected the number of exosomes in body fluids. Therefore, analysis of exosome levels may help in the early detection and diagnosis of lung diseases.^[16,17]^ Third, exosomes have the enormous therapeutic and prognostic potential for lung diseases: it has been shown that bone marrow mesenchymal stromal cell (MSC)-derived exosomes have great potential in the treatment of a variety of lung diseases, including pneumonia, inflammatory lung disease, acute respiratory distress syndrome, pulmonary hypertension, asthma, COPD, idiopathic pulmonary fibrosis, acute lung injury, and bronchopulmonary dysplasia.^[18]^ Related researchers have noted that bone marrow MSC-derived exosomes can affect immune, endothelial, and epithelial target cells of lung tissue and that their pathways are diverse,^[19,20]^ and that bone marrow MSC-derived exosomes can suppress pro-inflammatory responses and participate in lung tissue remodeling as well as oxidative stress.^[21]^ In the clinical treatment of respiratory diseases, bone marrow MSC-derived exosomes have the advantages of being smaller (approximately 100 nm), having a more comprehensive range of action, being inhaled as an aerosol, and treating airborne diseases, compared to MSCs.^[22]^

The bibliometric method has been utilized to measure scientific advancement in a variety of fields, as well as for systematic publication analysis.^[23]^ To date, bibliometric studies on exosomes have focused on cancer,^[24]^ bone and joint,^[25]^ cardiovascular diseases,^[26]^ ovarian cancer,^[27]^ and diabetes.^[28]^ Therefore, no bibliometric study has addressed exosomes and respiratory diseases. Therefore, a comprehensive summary of the latest research and hotspots in exosomes in lung diseases is necessary. Some new ideas can be provided to interested researchers. This bibliometric study aimed to explore the present state and hotspots of research on exosomes and respiratory diseases. Based on VOSviewer and CiteSpace software, relevant studies in this field were statistically analyzed. Firstly, the trend of annual publication volume in this field was systematically reviewed, and the classic research cases were listed among them. The national institutions, authors and their respective research directions, critical journals in the field, keywords, and subject headings that have made outstanding contributions to this field of research are then collated. Finally, we synthesize the above results to discuss the research hotspots and future development trends of exosomes in respiratory diseases to provide theoretical references for subsequent scholars and then promote the in-depth development of related research.

## 2. Methods

### 2.1. Data collection

The screening and evaluation of the relevant literature was carried out independently by 2 researchers. This research chose Web of Science Core Collection to be the data resource because of its excellent digital literature resource database, which is regarded by many academics as the most appropriate database for bibliometric research.^[29]^ The indexes Science Citation Index-Expended, Social Science Citation Index, Arts & Humanities Citation Index, Conference Proceedings Citation Index—Science, Conference Proceedings Citation Index—Social Sciences & Humanities, Emerging Sources Citations Index, current chemical reactions-expended, and Index Chemicus were chosen to guarantee that the search data was thorough and error-free. The search period was from the start of the WOS building until the end of May 4, 2022, and the search deadline was 22:00 on July 11, 2022. 2775 documents were found using the search strategy TS = (exosome OR exosomes OR exosomal) AND TS = (respiratory OR lung). The thesis, review paper, and online publication papers were chosen as the types of literature, and 2456 valid documents were included after excluding insufficient paperwork, including conference abstracts (196), editorial materials (42), book chapters (34) revisions (14), messages (11) conference proceedings papers (10) retracted articles (5) retracted publishers (4) data papers (2) news (1). Title, year of publication, journal, author, institution, nation, citation, and type of study were among the data that was extracted, exported, and saved in plain text format. Articles involving incomplete content were selectively excluded by the 2 researchers involved in the search, and unresolved disagreements were resolved by negotiation through a third party. The publication language was “English,” and the data were downloaded and analyzed by 2 researchers to ensure the data’s accuracy and the study’s reproducibility. Microsoft Excel and Endnote software were used to collect, organize and screen the literature data.

### 2.2. Data analysis

Pritchard introduced the concept of bibliometrics in the early 20th century. In 1969, bibliometrics was established as an independent subject.^[30]^ Nowadays, the fast growth of Internet computers in the big data information era has provided good conditions for the development of bibliometrics. By structuring and analyzing a large amount of information, it is possible to infer the research trends of disciplines or research fields over time, identify hot research, discover highly productive scholars and institutions, and show the general situation of current research fields.^[31]^ Literature co-citation also appears frequently in bibliometric analysis, and some studies have shown that visualization of co-citation analysis in bibliometrics can facilitate the interpretation of data, and the visual results can make further additions to the literature analysis and help to explore the inner association of information including authors, keywords, institutions, and countries.^[32]^

### 2.3. Statistical analysis

CiteSpace, built on scientometrics and data visualization analysis, was designed by Drexel University Professor Chaomei Chen in Java,^[33]^ which helps researchers to analyze the research field quantitatively and qualitatively and can improve our understanding of the research field. For example, CiteSpace computationally identifies critical points in the literature and highlights them in a visual network; by constructing a complex visual network of keywords or literature to highlight the development process and future trends of a specialty in a comprehensive way; by exploding words to indicate the research frontiers in the field under study at various periods.^[34]^ Leiden University’s Science and Technology Research Center created VOSviewer, a scientometric technology network analysis tool. VOSviewer can build views based on web-based literature data in keywords, journals, institutions, authors, etc. It can be displayed in up to 3 or 4 ways, such as label view, density view, cluster density view, and scatter view.^[35]^ It is characterized by graphical representation, which makes the data easy to understand, especially for some large maps.^[34]^ We used Microsoft Excel 2016 (Redmond, WA) to enumerate the obtained data and used Excel functions to present these data in tables or graphs. We used the following metrics to assess research results: (1) count, for example, number of publications, keyword co-occurrence network, and number of citations to the literature. (2) Betweenness centrality, (3) degree, (4) burst, and (5) citation tree-rings.

## 3. Results

### 3.1. General information

#### 3.1.1. The trend of publication outputs.

**Figure 1.**
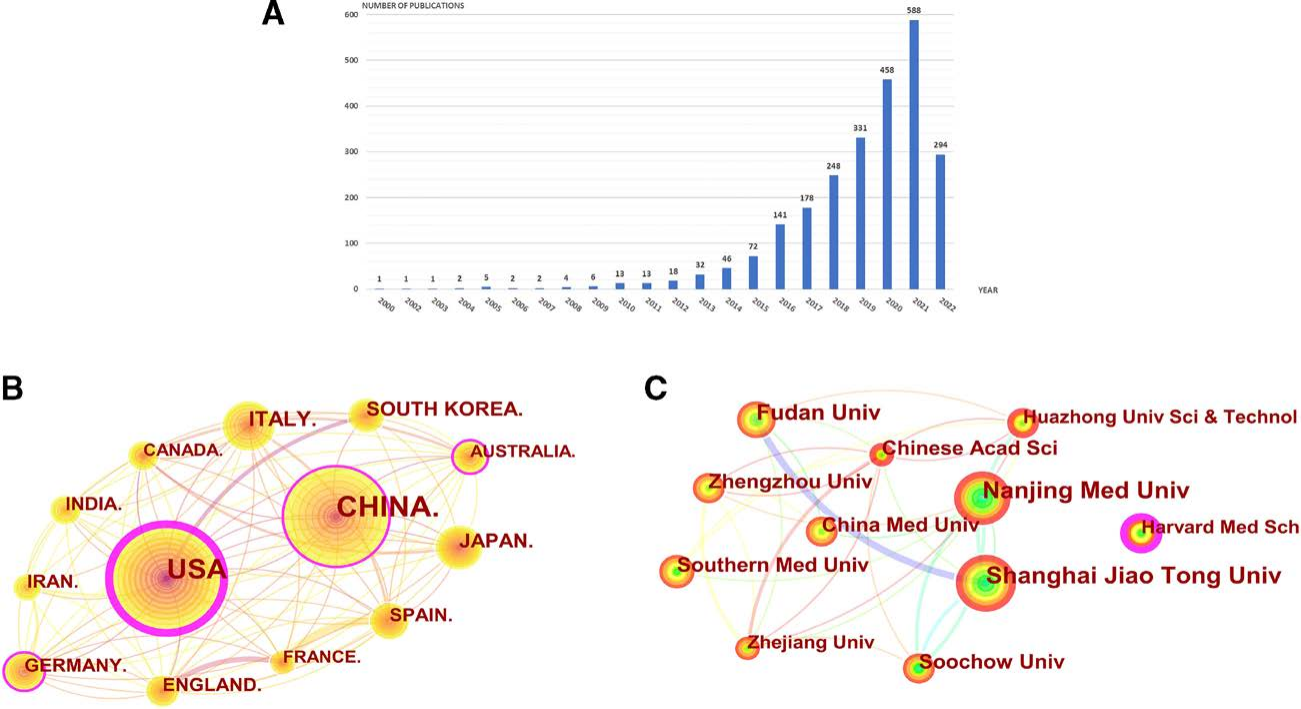
(A) Trends of exosomes published in respiratory/lung disease studies over the past 22 years. (B) CiteSpace visualization map of cooperation network of countries involved in exosomes in respiratory/lung disease. (C) CiteSpace visualization map of cooperation network of institutions involved in exosomes in respiratory/lung disease.

Figure 1A shows the number of publications and their trends in the field of exosomes involved in respiratory disease research from 2000 to 2022. Overall, the number of publications in this field has been on the rise, especially after 2012, when the number of publications showed a significant increase, surpassing 100 in 2016 and reaching a total of 588 by 2021 (Note: the search for 2022 publications ended on July 11.), indicating that more and more scholars are focusing on the study field. In contrast, before 2012, the number of articles issued increased generally, but the growth rate was sizable.

#### 3.1.2. Distribution of countries/regions and institutions.

The 2456 articles included in this study covered 2716 institutions in 76 countries. Every node symbolizes a nation/institution, and its dimension is proportionate to its article count. The more dense connections among nodes indicate more cooperation among nations/institutions. As shown in Figure 1B, Table 1, the highest number of articles was issued from China (43%), followed by USA (28%), Italy (6%), Japan (4%), and the 2 countries with the enormous amount of articles, China and USA, together account for 70% of the overall amount of articles. Both have the highest intermediary centrality, 0.41 and 0.09, respectively. With 4% of the total number of publications, Germany had the highest average citations per publication at 87.01. England, France, and Spain have the most robust cooperation among the many countries, and the 3 have the most expansive connection in the figure. As shown in Figure 1C and Table 2, the top 3 publishers are Shanghai Jiao Tong Univ, Nanjing Med Univ, and Fudan Univ, among which Shanghai Jiao Tong Univ has the highest average citations per publication, with 31.78. The highest intermediary centrality among all publishers is Harvard Med Sch with 0.23, which has a strong information control ability, but Harvard Med Sch has almost no cooperation with the top 10 publishers, while the top 10 publishers cooperate more frequently.

**Table 1:** The top 10 countries/regions with the largest number of articles.

Rank	Country	Documents	Citations	Average citation	Centrality
1	China	1053	25,731	24.43	0.09
2	USA	679	34,394	50.65	0.41
3	Italy	147	4635	31.53	0.08
4	Japan	109	8171	74.96	0.03
5	South Korea	102	2958	29	0.00
6	Germany	96	8353	87.01	0.1
7	Spain	82	6298	76.8	0.08
8	England	76	4110	54.08	0.08
9	Iran	71	1399	19.7	0.01
10	India	69	1620	23.48	0.06

**Table 2:** The top 10 institutions with the largest number of articles.

Rank	Organization	Documents	Citations	Average citation	Centrality
1	Shanghai Jiao Tong Univ	79	2511	31.78	0.07
2	Nanjing Med Univ	76	2204	29	0.10
3	Fudan Univ	52	1578	30.35	0.06
4	China Med Univ	47	913	19.43	0.02
5	Zhejiang Univ	41	953	23.24	0.06
6	Zhengzhou Univ	39	602	15.44	0.01
7	Soochow Univ	38	917	24.13	0.03
8	Southern Med Univ	38	827	21.76	0.07
9	Huazhong Univ Sci & Technol	35	730	20.86	0.01
10	Chinese Acad Sci	35	682	19.49	0.08

#### 3.1.3. Contribution of journals.

**Figure 2.**
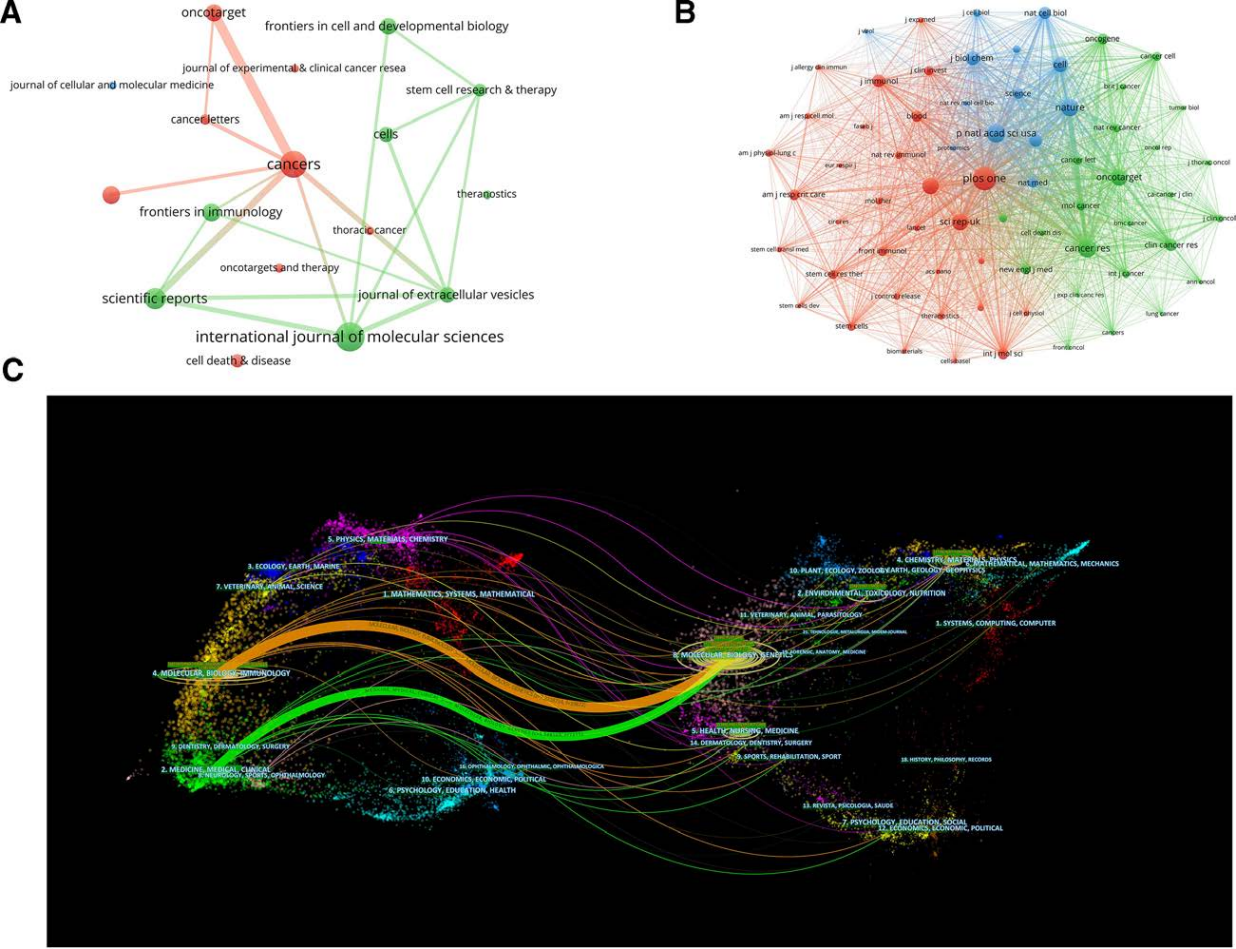
(A) VOSviewer visualization map of journals involved in respiratory/lung disease exosomes. (B) VOSviewer visualization map of Co-citation of Cited Journals involved in exosomes in respiratory/lung disease. (C) The dual-map overlay of journals on exosomes in respiratory/lung disease.

The study involved 2456 papers from 738 journals. International Journal of Molecular Sciences had the most pertinent publications (2.69%), followed by Cancers (2.44%), and Scientific Reports (1.95%), according to Figure 2A, Table 3. Journal of Extracellular Vesicles has the most impact factor within the best 10 journals regarding the number of publications, at 17,337, followed by Cell Death & Disease at 9684. Journal of Extracellular Vesicles has the greatest average number of citations per article (83.83) within the top 10 journals regarding the quantity of articles published, showing that it has some influence in the area of exocrine research in respiratory illnesses. It is a key publication in this area.

**Table 3:** The top 10 journals with the largest number of articles.

Grade	Source	Documents	Citations	Average citation	IF (2021)	JCR
1	International Journal of Molecular Sciences	66	1273	19.29	6.208	Q1
2	Cancers	60	592	9.87	6.575	Q1
3	Scientific Reports	48	1817	37.85	4.996	Q1
4	Frontiers in Immunology	40	819	20.48	8.786	Q1
5	Frontiers in Oncology	39	607	15.56	5.738	Q2
6	Oncotarget	38	2559	67.34	4.345	Q2
7	Frontiers in Cell and Developmental Biology	37	322	8.7	6.081	Q1
8	Journal of Extracellular Vesicles	35	2934	83.83	17.337	Q1
9	Cells	34	673	19.79	7.666	Q2
10	Stem Cell Research & Therapy	29	1247	43	8.079	Q1

#### 3.1.4. Contribution of scholars.

**Figure 3.**
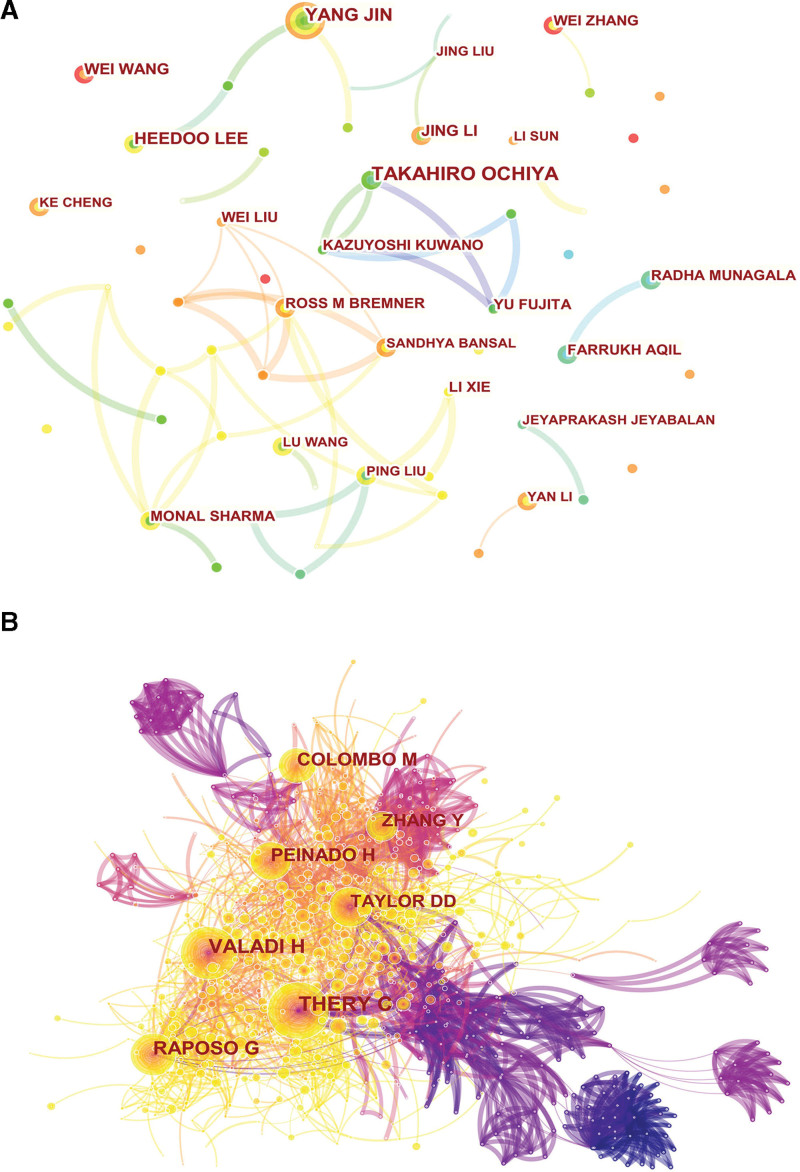
(A) CiteSpace visualization map of authors’ co-authorship in respiratory/lung disease exosomes. (B) CiteSpace visualization map of Co-citation of Cited Authors involved in exosomes in respiratory/lung disease.

14,568 researchers were involved in publishing articles related to exosomes in respiratory diseases. The resulting data are included in Table 4. Among the highly productive authors, Takahiro Ochiya had the highest number of articles (21 articles), with 2134 citations and an average of 102.62 citations per article, while Yang Jin (20 articles) had the second highest number of citations (747 citations), with a significantly lower number of citations than the former, with an average of 37.35 citations per article. It is worth mentioning that Stella Kourembanas, who is ranked seventh in total publications, has an average of 102.42 citations per article, which is almost the same as Takahiro Ochiya. As depicted in Figure 3A, exists a network of interaction and cooperation between researchers within the study area, where each node represents an author, and the larger the node, the more articles the author has published, and the lines represent collaboration among authors, with different colors indicating different clusters of close partnership. Kazuyoshi Kuwano, and Yu Fujita, are a collaborative cluster, Yang Jin and Heedoo Lee is a collaborative cluster, and Thalachallour Mohanakumar and Sandhya Bansal are a collective group.

**Table 4:** - The top 10 scholars with the largest number of articles.

Rank	Author	Documents	Citations	Average citation
1	Ochiya, Takahiro	21	2134	102.62
2	Jin, Yang	20	747	37.35
3	Zhang, Wei	17	552	32.47
4	Li, Jing	14	254	18.14
5	Mohanakumar, Thalachallour	13	133	10.23
6	Bansal, Sandhya	13	120	9.23
7	Kourembanas, Stella	12	1229	102.42
8	Fujita, Yu	12	629	52.42
9	Kuwano, Kazuyoshi	12	629	52.42
10	Lee, Heedoo	12	614	51.17

#### 3.1.5. Neighbourhoods involved.

The study involved 2456 publications, which were analyzed by CiteSpace visualization of the relevant domains (Fig. 6B), resulting in 96 nodes and 484 linked lines; each node represents a domain, with the node’s magnitude proportional to the frequency with which the domain appears in the literature, and the wider the purple circle outside the node tree wheel indicating the higher the mediated centrality of the domain. Oncology appears most frequently at 507 times, followed by Cell Biology (433 citations) and Biochemistry & Molecular Biology (305 citations), indicating that these fields are prevalent in studying exocrine and respiratory diseases. The field with the highest intermediary centrality in Biochemistry & Molecular Biology, with a value of 0.34, followed by Engineering (0.28) and Cell Biology (0.24), indicating that these fields play an essential linking role in other fields and are more critical.

**Figure 4.**
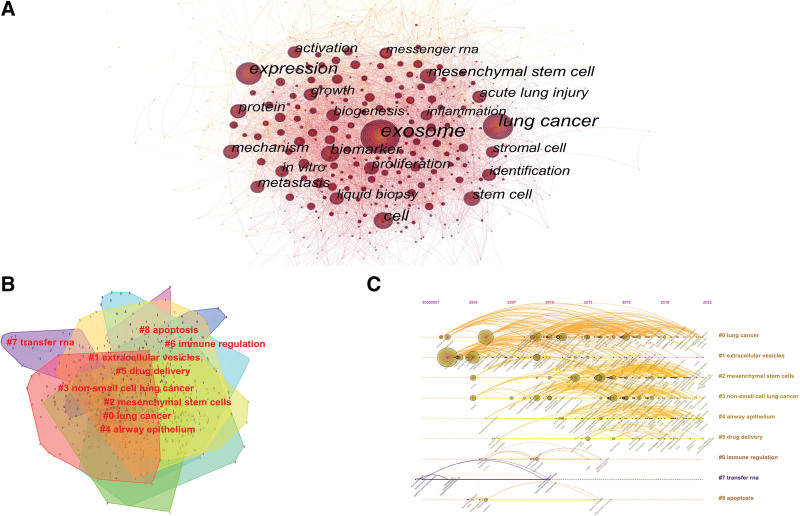
(A) CiteSpace visualization map of keyword co-occurrence in exosomes in respiratory/lung disease. (B) CiteSpace visualization map of keyword cluster involved in exosomes in respiratory/lung disease. (C) CiteSpace visualization map of keyword timeline view involved in exosomes in respiratory/lung disease.

**Figure 5.**
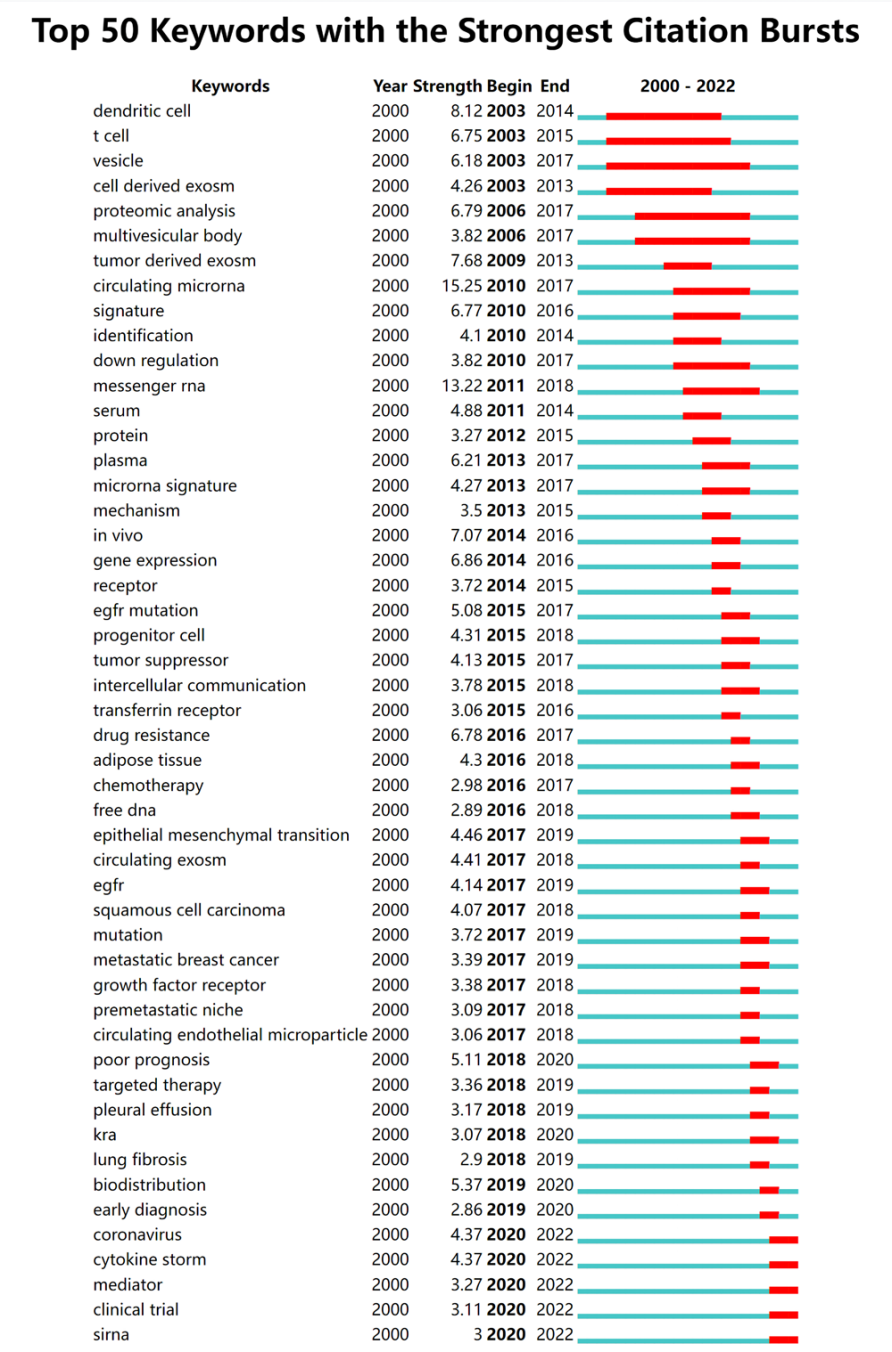
The top 50 keywords with the most robust citation bursts.

### 3.2. The analysis of keyword and term

#### 3.2.1. The analysis of keyword co-occurrence.

7888 keywords were involved in the 2456 articles used in the study, and 679 keywords were obtained after integration by CiteSpace data processing, using the keywords as nodes and visual graph analysis to produce 679 nodes and 5173 connecting lines. The dimension of the nodes shows the occurrence rate of the keywords, the connecting lines represent the intensity of association, and the thickness of the connecting lines denotes the amount of keyword appearances (Fig. 4A). The top 20 keywords in frequency are shown in Table 5, among which the highest frequency is exosome with 1265 occurrences, followed by lung cancer (636 occurrences) and expression (456 occurrences). The highest intermediate centrality other than exosome was lung cancer, and dendritic cell, both with 0.10, followed by expression with 0.07. Table 5 shows that the keywords: exosome, lung cancer, expression, activation, in vitro, mesenchymal stem cell, mechanism, and cell were found in the keyword frequency top 20 and keyword mediated centrality top 20.

**Table 5:** Top 20 keywords related to exosomes in respiratory disease.

Grade	Keyword	Occurrences	Grade	Keyword	Centrality
1	Exosome	1265	1	Exosome	0.14
2	Lung cancer	636	2	Lung cancer	0.1
3	Expression	456	3	Dendritic cell	0.1
4	Cell	263	4	Expression	0.07
5	Mesenchymal stem cell	204	5	Activation	0.06
6	Biomarker	192	6	Cell derived exosm	0.06
7	Protein	156	7	In vivo	0.06
8	Metastasis	148	8	In vitro	0.05
9	Proliferation	148	9	Tumor derived exosm	0.05
10	Stem cell	148	10	Mesenchymal stem cell	0.04
11	Mechanism	146	11	Mechanism	0.04
12	Activation	142	12	Messenger RNA	0.04
13	Biogenesis	137	13	Lung	0.04
14	Acute lung injury	136	14	Down regulation	0.04
15	Growth	134	15	T cell	0.04
16	In vitro	134	16	Endothelial cell	0.04
17	Liquid biopsy	132	17	Cancer cell	0.04
18	Identification	125	18	Cancer patient	0.04
19	Stromal cell	124	19	Human pm scl	0.04
20	Inflammation	100	20	Cell	0.03

#### 3.2.2. The analysis of keyword cluster.

The 679 keywords were clustered by the CiteSpace software used the LSI algorithm to create 9 clusters of varying sizes and hues, with significant overlaps between clusters indicating close links between clusters (Fig. 4B). One of them, #0 lung cancer, had the most terms (152), followed by #1 extracellular vesicles and #2 mesenchymal stem cells, each with 119 keywords. The key phrases in each cluster were summarized (Table 6). The study of exosomes in respiratory illnesses is thought to concentrate on lung cancer, mesenchymal stem cells, airway epithelial cells, drug delivery system, immunological control, and transport RNA, among which lung cancer, extracellular vesicles, and mesenchymal stem cells are prominent.

**Table 6:** - Details of clusters.

Label	Size	Year	The main keywords included (sorted from high to low frequency)
#0 lung cancer	152	2014	Lung cancer, cell, biomarker, liquid biopsy, identification, messenger rna, rna, cell lung cancer, plasma, circulating tumor cell,
#1 extracellular vesicles	119	2017	Bronchoalveolar lavage fluid, tgf beta, exposure, immunity, antigen, multivesicular body, acute respiratory syndrome, bronchoalveolar lavage, cell migration, dendriticcell
#2 mesenchymal stem cells	119	2009	Mesenchymal stem cell, stem cell, acute lung injury, in vitro, stromal cell, inflammation, therapy, lung injury, mesenchymal stromal cell, differentiation
#3 non-small cell lung cancer	111	2016	Metastasis, proliferation, growth, progression, invasion, non-small cell lung cancer, colorectal cancer, down regulation, migration, pathway
#4 airway epithelium	73	2017	Lung, receptor, endothelial cell, epithelial cell, nf kappa b, pathogenesis, phenotype, pulmonary fibrosis, induction, antibody
#5 drug delivery	35	2014	Delivery, drug delivery, nanoparticle, biodistribution, brain, gene therapy, cancer therapy, blood brain barrier, sirna, paclitaxel
#6 immune regulation	28	2007	Angiogenesis, in vivo, cancer cell, melanoma, growth factor receptor, induce, monocyte, peptide based vaccine, activated platelet, clendritic cell
#7 transfer RNA	19	2002	Carcinoma cell, systemic lupus erythematosus, human pm scl, interstitiallung disease, histonedeacetylase, in situ, ny eso 1, idiopathicinflammatory myopathy, bmi 1oncoprotein
#8 apoptosis	14	2005	Apoptosis, adhesion, membrane, alpha, factor binding protein, surface, inflammatory monocyte, tumor necrosis factor, generation, human urine

Based on the clustering of keywords, the keyword timeline view (Fig. 4C) was drawn using CiteSpace software, which can objectively reflect the changes in the topics contained in each subcluster and the development process and stage hotspots of overall respiratory disease and exosome research from the time dimension. The timeline diagram was analyzed in the following aspects: (1) The earliest time cluster was *#7 transfer RNA*, and the first keyword interstitial lung disease appeared in 2000, in the first paper Update on myositis-specific and myositis-associated autoantibodies, followed by clusters *#0 lung cancer, #1 extracellular vesicles*, the first keywords appeared in 2001, respectively, identification, exosome. (2) Cluster *#0 lung cancer* showed a significant increase in the number of research results in 2008 and will remain so until 2022, while scholars have valued cluster #1 extracellular vesicles since its appearance in 2001; Similarly, clustering *#2 mesenchymal stem cells* first keyword in vitro appeared in 2004, and its findings started to increase significantly in 2009. (3) Cluster *#7 transfer RNA* started to have a longer keyword interval from 2002, and the attention of its research started to decrease in 2010, while clusters *#6 immune regulation* and *#8 apoptosis* started to decrease from 2009 and 2005, respectively. (4) The keyword chronology and labels form an element, and the size of the keyword chronology in the figure depends on the frequency of the keywords. Exosomes in respiratory diseases had not received significant attention before 2001, while from 2001 to 2022, this research has increased significantly; as can be seen from the figure, a large number of iconic keywords emerged during this period, such as exosome, expression, lung cancer, cell, mesenchymal stem cell, biomarker, protein, metastasis, proliferation, stem cell, mechanism, activation, biogenesis, acute lung injury, growth, in vitro, liquid biopsy, identification.

#### 3.2.3. The analysis of keywords prominence.

Keyword emergence is a way to understand the hot spots and frontiers of research in the field by analyzing the keywords that proliferate in a short period and suddenly become hot spots, thus being noticed by the academic community, and to predict the future research trends for the scholars’ further in-depth research. Overall, there is a more even distribution of exosome-related terms in respiratory illness research, and Figure 5’s intensity of burst words is all over 2.8. Vesicle is the burst cycle that lasts the longest, from 2003 to 2017, followed by t cell (13 years) and dendritic cell (12 years). Circulating microRNA had the highest burst intensity (15.25), followed by messenger RNA (13.22), dendritic cells (8.12), and tumor-derived exosomes (7.68). The last 3 years have seen a more significant outbreak for the coronavirus, cytokine storm, mediator, clinical trial, and siRNA, which are expected to get more attention in the future.

#### 3.2.4. The analysis of term.

**Figure 6.**
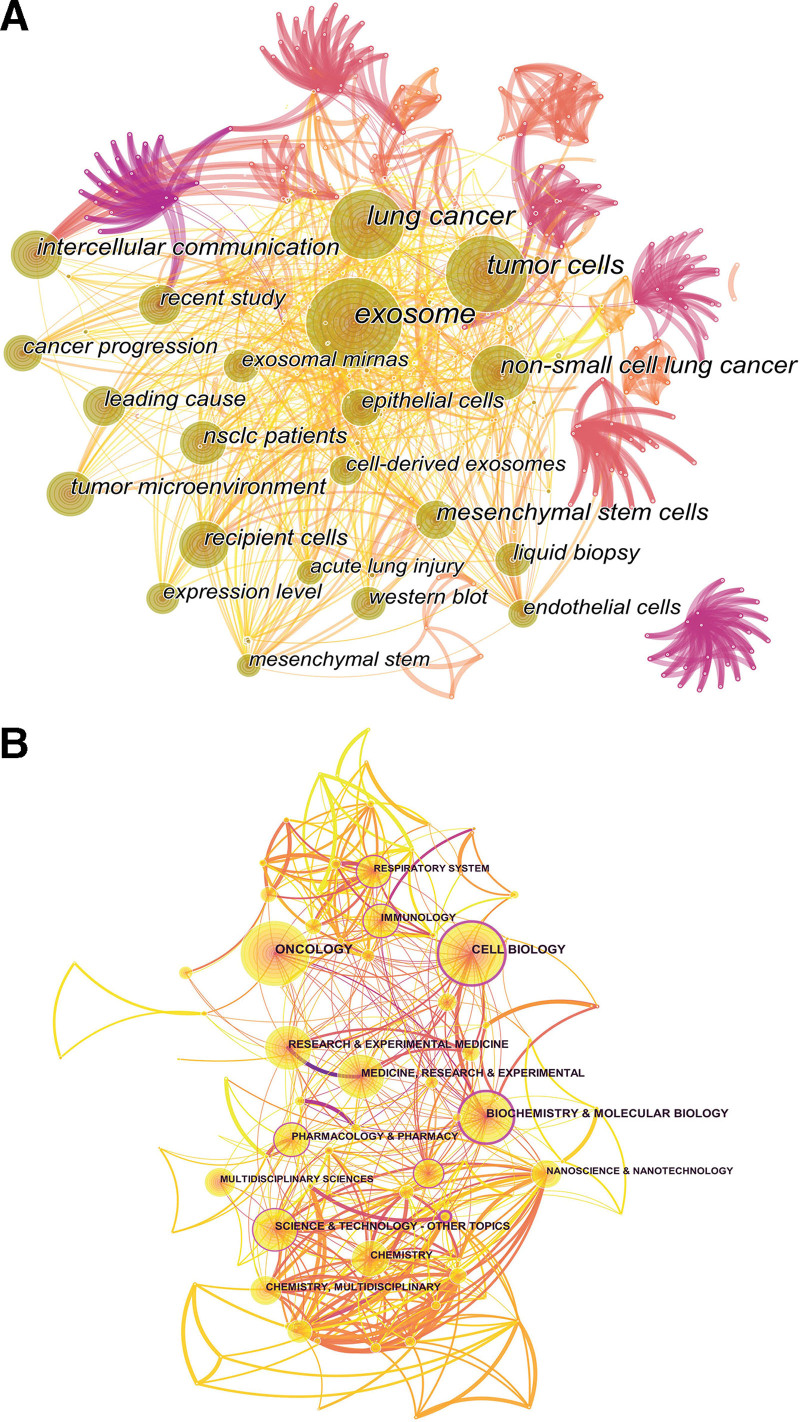
(A) CiteSpace visualization map of the term involved in exosomes in respiratory/lung disease. (B) CiteSpace visualization map of citation fields involved in respiratory/lung disease exosomes.

Through Citespaces, we mapped the visualization of exosomes and respiratory disease research themes (Fig. 6A), forming 827 nodes and 4925 connected lines to the map; we concluded that the distribution of exosomes in respiratory disease theme words are mainly in lung cancer, non-small cell lung cancer, mesenchymal stem cells, intercellular communication, exosomal miRNAs, epithelial cells, dendritic cell exosomes, receptor cells, acute lung injury, liquid biopsy, protein blotting, and endothelial cells.

### 3.3. Co-citation analysis

#### 3.3.1. Co-citation of cited references.

**Figure 7.**
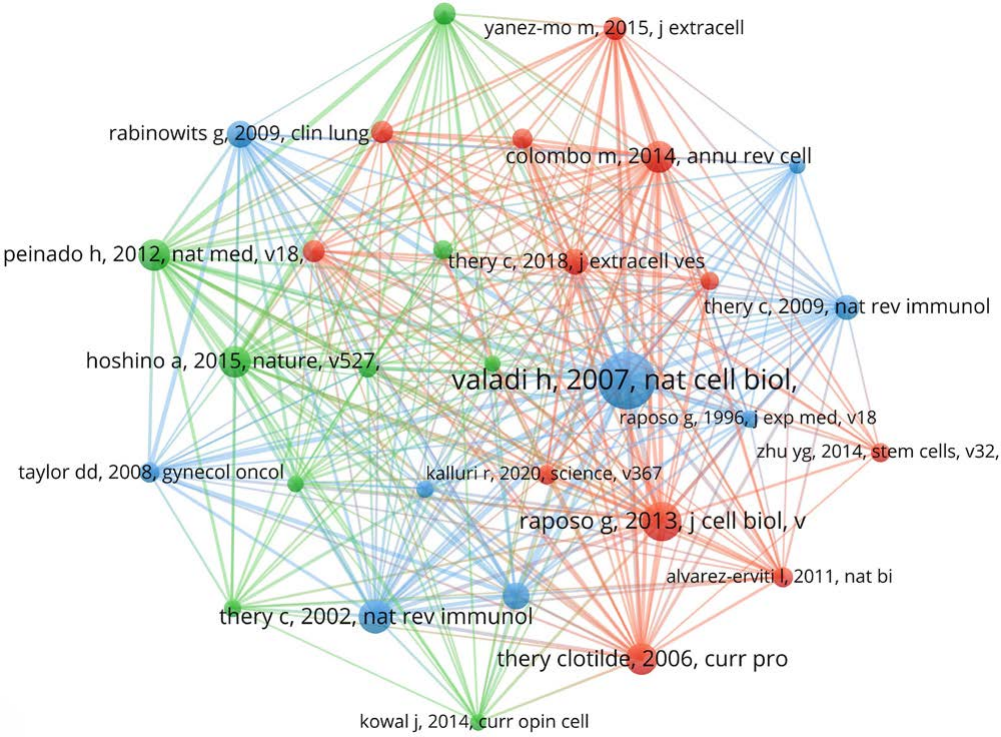
CiteSpace visualization map of co-citation of cited references involved in exosomes in respiratory/lung disease.

**Figure 8.**
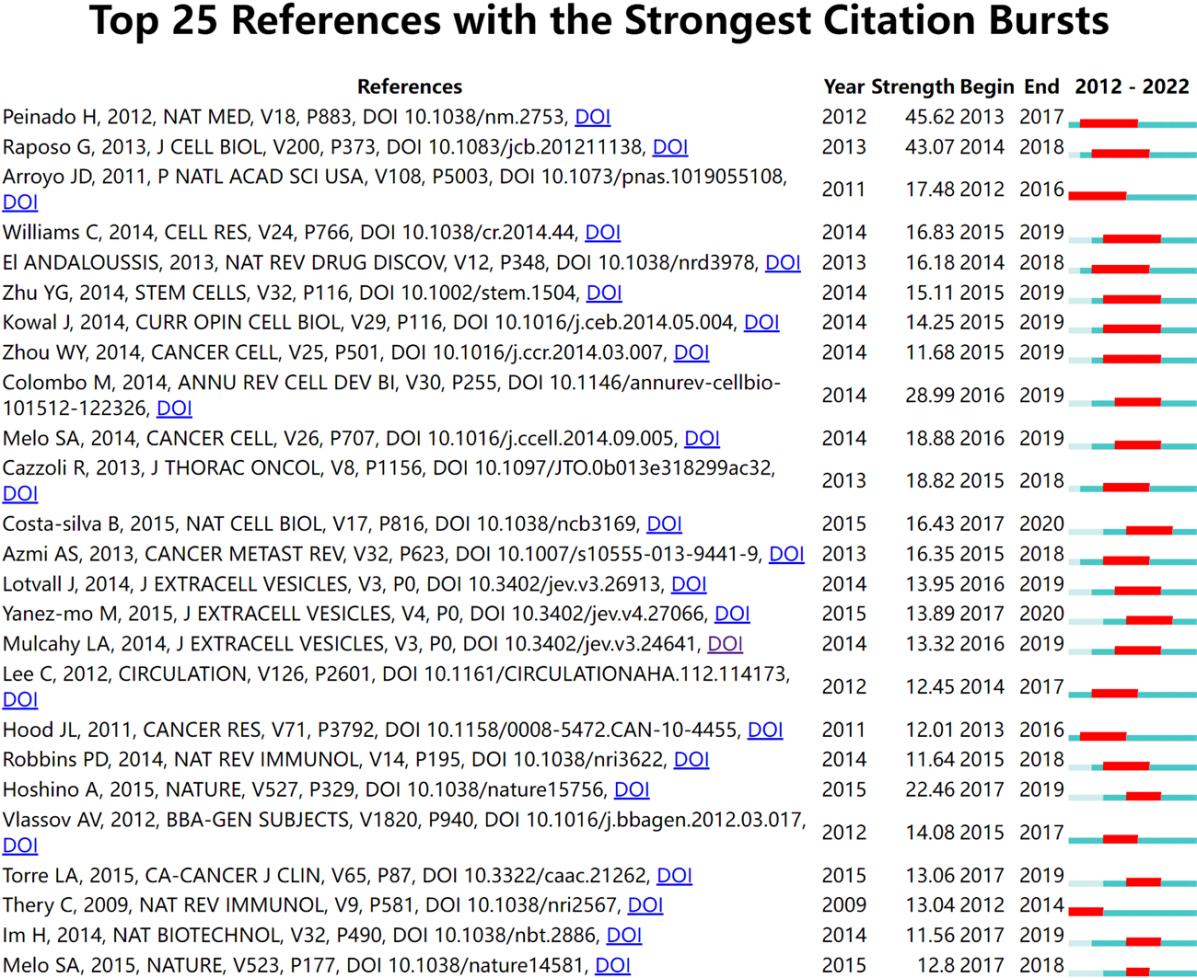
The top 25 references with the most robust citation bursts.

In this study, the total number of references cited in the 2456 articles was 97,983, and among the 97,983 cited articles, 30 articles were cited ≥100 times, and these 30 articles were analyzed by using VOSviewer to visualize the co-citation relationship (Fig. 7), and each node represented a cited article. The 30 co-citations were divided into 3 clusters and represented by 3 colors. The top 11 most frequently cited literature on exosomes in respiratory diseases (Table 7) were also listed; the top 3 references were referenced over 248 occasions. Exosome-Mediated Transfer of mRNAs and Micrornas is a Novel Mechanism of Genetic Exchange Between Cells was its most frequently referred to piece of literature (475 times), with an outbreak intensity of 14.57 and an outbreak date of 2008. The paper demonstrates that exosomes include mRNA and microRNA and could be transported into other cells to function.^[36]^ As shown in Figure 8, the topic with the highest outbreak intensity among references was Melanoma Exosomes Educate Bone Marrow Progenitor Cells Toward a Pro-Metastatic Phenotype Through Met at 45.62, starting in 2013 and ending in 2017; the article describes the effect of melanoma exosomes on bone marrow progenitor cells,^[37]^ indicating that the content of the article was firmly pushed by scholars within the period between 2013 to 2017. As shown in Table 7, the top 11 most frequently cited publications accounted for 4 studies on exosomes in oncology, among which Rabinowits G, Exosomal MicroRNA: A Diagnostic Marker for Lung Cancer clearly describes the clinical diagnostic value of exosomes in lung cancer.

**Table 7:** Top 11 co-cited references related to exosomes in respiratory disease.

Grade	Cited reference	Year	Citations
1	Valadi H: Exosome-Mediated Transfer of MRNAs and Micrornas is a Novel Mechanism of Genetic Exchange Between Cells	2007	475
2	Raposo G: Extracellular Vesicles: Exosomes, Microvesicles, and Friends	2013	299
3	Thery C: Exosomes: Composition, Biogenesis and Function	2002	249
4	Thery C: Lotildeisolation and Characterization of Exosomes from Cell Culture Supernatants and Biological Fluids	2006	233
5	Colombo M: Biogenesis, Secretion, and Intercellular Interactions of Exosomes and Other Extracellular Vesicles	2014	227
6	Peinado H: Melanoma Exosomes Educate Bone Marrow Progenitor Cells Toward a Pro-Metastatic Phenotype Through Met	2012	227
7	Hoshino A: Tumour Exosome Integrins Determine Organotropic Metastasis	2015	226
8	Skog J: Glioblastoma Microvesicles Transport RNA and Proteins that Promote Tumour Growth and Provide Diagnostic Biomarkers	2008	191
9	Rabinowits G: Exosomal MicroRNA: A Diagnostic Marker for Lung Cancer	2009	186
10	Thery C: Minimal Information for Studies of Extracellular Vesicles 2018 (Misev2018): A Position Statement of the International Society for Extracellular Vesicles and Update of the Misev2014 Guidelines	2018	177
11	Thery C: Membrane Vesicles as Conveyors of Immune Responses	2009	172

#### 3.3.2. Co-citation of cited journals.

The 979,883 references were from 7045 journals, and VOSviewer was utilized to limit the smallest amount of citations of journals to 500 in order to find the 68 journals with the highest number of citations and to visualize the relationship of periodical co-citations into a visual graph (Fig. 2B). As can be seen from the figure, the network co-citation map of journals is divided into 3 significant clusters represented by 3 colors, and every node symbolizes a journal, as well as the journal’s magnitude reflects how many times it has been cited. The top 5 journals in terms of citations are Plos One (3882 citations, IF: 3.752), Proceedings of the National Academy of Sciences of the United States of America (2792 citations, IF: 12.78), Cancer Research (2770 citations, IF: 12.78), Nature (2667 citations, IF:69.504), Journal of Extracellular Vesicles (2470 citations, IF:17.337).

Combining Figure 2A and B, in order to further analyze the journals involved in indexing 2456 documents and 97,983 cited documents, we used CiteSpaces to create a biplot overlay analysis graph of journals (Fig. 2C), in which the left is the citation graph and the right is the cited graph. The 2 graphs comprehensively show the coming and going of citations through curves (citation linkage), and the colors of the curves are mainly yellow, green, and purple. The thickness of the traces is proportional to the number of citations. The labels in the graphs refer to the discipline or field corresponding to the articles published in the journals, and the clustering centers of the corresponding journals are the main ones. The literature on exocrine and respiratory diseases in 4. Molecular, Biology, Immunology 2. Medicine, Medical, and Clinical are influenced by the dominant field of 8. Molecular, Biology, Genetics, and a specific research field shift.

#### 3.3.3. Co-citation of cited authors.

979,883 references covering 63,881 authors. The criteria for the lowest amount of citations that an author must have was established at 200 using VOSviewer. This was done in order to acquire the top 20 writers who were cited the most and to show the relationships between authors who were cited together (Fig. 3B), where every node symbolizes a writer. Node’s magnitude corresponds to the number of citations for that writer. 20 co-cited authors were divided into 2 clusters and represented by 2 colors. The most cited authors are Clotilde Théry, and Hadi Valadi, both with 739 citations, followed by Raposo, G (477 citations), Peinado, H (310 citations), and Zhang, Y (292 citations).

## 4. Discussion

### 4.1. General information

Of the 2456 articles retrieved from the WOS database from November 1, 2000, to July 11, 2022, at 22:00, the first systematic discussion of exosomes and respiratory diseases appeared in 2003, which indicated that exosomes are present in BALF of healthy individuals and may play a regulatory role in the local immune defense of the lung,^[38]^ and in 2004 exosomes were isolated from the pleural effusions of lung cancer patients.^[38]^ Throughout the period, there was just a marginal drop in the total number of publications seen in 2005 to 2006, while in 2005, studies focused on exosomes and lung cancer,^[39–41]^ and related lung cancer studies as a starting point showed an overall increasing trend in subsequent studies and reached a maximum in 2021 (85 articles). In 2007, related scholars discovered that exosomes derived from BALF of bovine mycoplasma-infected mice promote intercellular interaction during the immune response to intracellular infections,^[42]^ which stimulated the study of exosomes in immune and inflammatory responses in respiratory diseases.^[43]^ In general, the preliminary studies on exosomes in respiratory diseases from 2000 to 2012 mainly focused on the isolation and extraction of exosomes and their role in lung cancer, lung inflammatory diseases, and lung immune response; the shortcoming is that the overall number of literature appears to be low, the growth trend is not apparent, and numerous aspects of research are still in the nascent stage, such as exosomal MicroRNA,^[44]^ exosomes in bronchus,^[45]^ etc. However, the research at that stage laid a solid basis for future progress in this domain. Since 2012, the relevant literature in this field has increased significantly. The number of exocrine articles on various aspects of respiratory diseases has increased rapidly in the past 3 years, indicating that research in this direction has been moving toward a more mature state.

In general, there are collaborative networks between countries, the width and strength of their collaboration are satisfactory, and the exchange of research results is abundant, whereas, in terms of institutions, the major Chinese publishers such as Shanghai Jiaotong University and Nanjing Medical University are limited to domestic cooperation and have few academic exchanges with foreign countries, which significantly limits the development of exosomes in this field of respiratory diseases. Therefore, it is recommended that domestic institutions and teams should communicate and learn more from foreign intermediaries with higher centrality of publication while maintaining the advantages of their research areas. The first publication from Chinese institutions in the study of exosomes in respiratory diseases was in 2008. There have been more significant growth since 2010, with 285 publications in 1 year in 2021, and their main research directions are distributed in mesenchymal stem cell-derived exosomes and acute lung injury,^[46,47]^ exosomal tumor RNA and lung metastasis of tumors,^[48–51]^ and exosomal miRNAs and non-small cell lung cancer,^[52–54]^ and exosomes and lung cancer.^[55,56]^ In contrast, the first relevant literature was published by US institutions in 2000, and there was a significant rise from 2011, with 126 articles published in 1 year in 2021, focusing on the following research areas: exosomes and lung cancer,^[57,58]^ exosomal microRNA and lung cancer,^[59]^ exosomes and non-small cell lung cancer,^[60]^ MSC-derived exosomes, and acute lung injury, pneumonia.^[61–65]^

By analyzing the corresponding authors in the literature, we derived the core authors in the field of exocrine and respiratory disease research and the focus of their respective research directions.

Ochiya, Takahiro (H-index:69): President of the Japan Society for Extracellular Vesicles (JSEV). His research interests include siRNA, and microRNA-based cancer stem cell therapies and exosome research in the field of cancer. His previous exosome research has focused on breast cancer,^[66–69]^ Alzheimer disease,^[70]^ prostate cancer,^[71]^ and liver cancer.^[72]^ Studies on the involvement of exosomes in respiratory diseases have been more cursory; for example, he proposed that RNAi can perform gene medicine therapeutic interventions against various human diseases such as infections, respiratory diseases, and cancer^[73]^; exosomes of specific cell types could be used as a novel means of anticancer therapy, immunomodulation, regenerative therapy, and drug transport,^[74]^ which did not generate in-depth studies. In the last few years, Prof Ochiya Takahiro has tended to study exosomes in respiratory diseases, mainly in the treatment of inflammatory lung diseases with MSC-derived exosomes,^[71]^ the effect of exosomes on the pulmonary immune system,^[75]^ the role of exosomes from fibroblasts in pulmonary fibrosis,^[76,77]^ and the potential of exosomes in the treatment of non-small cell lung cancer,^[78]^ the significance of urinary exosome microRNA detection for the earlier detection of pulmonary cancer,^[79]^ and the effect of exosomes secreted by senescent cells on age-related lung diseases.^[80]^ By collating and analyzing the relevant papers of Prof Ochiya, and Takahiro, it can be found that their research on exosomes in respiratory diseases is more extensive, involving pulmonary fibrosis, COPD, and non-small cell lung cancer. However, the main research direction is exosomes and lung cancer.

Kourembanas, Stella (H-index: 27): Works in the Department of Pediatrics, Division of Neonatal Medicine, Harvard Medical School Children’s Hospital, with research interests in neonatal and pediatric respiratory diseases.^[81]^ The following studies related to exosomes were compiled by Dr Kourembanas, Stella: MSCs exosomes improve experimental bronchopulmonary dysplasia and restore lung function through macrophage immunomodulation,^[71]^ MSCs extracellular vesicles ameliorate allergic airway inflammation induced by Aspergillus mycelium extract in mice,^[71]^ MSCs exosomes are significant carriers of immunomodulatory and therapeutic effects in animal models of lung disease,^[82]^ Exosome therapy in neonatal lung injury,^[83]^ MSCs exosomes as gatekeepers in pulmonary hypertension,^[84]^ The MSC-derived exosomes prevent and reverse experimental pulmonary fibrosis by modulating monocyte phenotype,^[85]^ MSCs extracellular vesicles can recover pulmonary structures in neonatal lung injury models.^[86]^ The analysis revealed that the study by Dr Kourembanas, Stella focused on the significance of MSC exosomes in respiratory diseases with a focus on pulmonary fibrosis and pulmonary hypertension.

The above analysis of co-cited literature and co-cited authors shows that among the top 11 most frequently cited references, articles by author Clotilde Théry accounted for 4 articles. Among the top 10 authors who received citations (Table 7), the author with the highest number of citations was Clotilde Théry, with 739 citations. Thus, the content of scholar Clotilde Théry’s research has profoundly impacted the existing literature on exosomes and respiratory diseases, which is valuable for his and related scholars’ research. Scholar Clotilde Théry (H-index: 49) has a long-standing interest in the role of exosomes (vesicles) in immunology and tumor^[87–89]^ and has established a research team on “Exosomes and tumor growth” at the Curie Institute. The “Exosomes and tumor growth” research team was established at the Curie Institute. Scholar Hadi Valadi (H-index:27) has worked on the relationship between exosomes and RNA^[71]^ and is currently trying to insert therapeutic RNA into exosomes and transfer it to other cells.^[90]^ Scholar Graça Raposo (H-index:88) has focused on extracellular vesicles^[91]^ and received the Special Achievement Award from the International Society for Extracellular Vesicles in 2018.

### 4.2. The hotspots and frontiers

The analysis of keywords can reflect the trend of theme evolution and study hotspots in particular study areas within a particular time frame. Through the above analysis of keyword co-occurrence, keyword clustering, keyword emergence, and theme words, we discuss and summarize the research hotspots of exosomes in respiratory diseases as follows.

#### 4.2.1. Exosomes and lung cancer.

Nowadays, lung cancer still accounts for a large proportion of deaths from tumors worldwide, and with the growing body of knowledge about exosomes and oncology,^[92]^ more and more researchers are concentrating on the role of exosomes in lung cancer, particularly on the effects of various source types of exosomes on the development and metastasis of lung cancer. Ratajczak, MZ et al investigated the mechanism of platelet-derived exosomes in lung cancer development and metastasis and found that platelet-derived exosomes promoted the multiplication of some lung cancer cell lines by stimulating the expression of related proteases; meanwhile, in a mouse model, it was found that lung cancer mice injected with platelet-derived exosomes had significantly more metastatic foci in the lungs than mice not injected with platelet-derived exosomes. This demonstrates the critical role of platelet-derived exosomes in the progression as well as migration of lung cancer.^[71]^ The article was published in the International Journal of Cancer (IF: 7.316) and has been cited 550 times. In addition, Chinese scholars Yang Wen and Wang Hongyang showed that tumor-derived exosomes could induce cancer-associated fibroblast activation and thus promote lung metastasis of hepatocellular carcinoma,^[71]^ published in Nature Communications (IF: 17.694) and cited 450 times.

Exosomes are utilized to diagnose and prognosticate non-small cell lung cancer, which accounts for 85% of lung tumor occurrences. Non-small cell lung cancer is divided into adenocarcinoma (40%) and squamous carcinoma (25%), emphasizing early detection, diagnosis, and treatment. Xie, Deyao, Wenzhou Medical University, China, found that miR-181-5p and miR-30a-3p are particular to adenocarcinoma and miR-10b-5p and miR-15b-5p are particular to squamous carcinoma.^[71]^ This literature was obtained from Clinical Cancer Research (IF: 13.801), cited: 300 times. Similarly, Jiang, Yiguo, a scholar from Guangzhou Medical University, suggested that exosomes could transfer cyclic RNA circSATB2 and facilitate the multiplication as well as metastasis of non-small cell lung cancer and induce abnormal proliferation of bronchial epithelial cells. Just because the cyclic RNA circSATB2 can be expressed explicitly in the exosomes of serum from lung cancer patients, its exosomes have the potential to be used as a diagnostic marker for non-small cell lung cancer.^[93]^ This literature is from Molecular Cancer (IF: 41.444), cited: 106 times.

#### 4.2.2. Mesenchymal stem cell-derived exosomes and lung inflammation.

Mesenchymal stem cells are adult stem cells of mesodermal origin that can differentiate into a variety of mesenchymal tissues and can induce peripheral immune tolerance and affect damaged tissues in vivo, inhibiting the release of pro-cytokines and promoting the survival of damaged cells, thus coming to immune reconstitution.^[94]^ In a study of mesenchymal stem cell innate immune regulation, the American scholar Ortiz, Luis A. found that bone marrow MSC-derived exosomes in a designed lung injury model reduced the accumulation of neutrophils and lymphocytes in BALF and possessed the ability to suppress inflammation in the lung.^[71]^ This article was published in Nature Communications (IF: 17.694) Cited: 502 times.

Lee, Jae W, a scholar at the University of California, by exploring the effects of human MSC-derived exosomes on lung inflammation, protein permeability, and bacterial clearance after severe bacterial pneumonia found that MSC-like exosomes were able to reduce the influx of inflammatory cells, cytokines, proteins, and bacteria, thus demonstrating that MSC-derived exosomes possess MSC-like immunomodulatory functions in severe bacterial pneumonia.^[71]^ The article was published in the American Journal Of Respiratory And Critical Care Medicine(IF: 30.528) and cited: 287 times. COVID-19-infected pneumonia in Wuhan, China, in 2019, a related scholar explored the therapeutic role of MSC-derived exosomes in COVID-19 and indicated that exosomes from allogeneic bone marrow MSCs could attenuate cytokines and reconstitute the immune system, and were a potential drug for the treatment of COVID-19.^[71]^

#### 4.2.3. MiRNA in exosomes as a biomarker in respiratory diseases.

MiRNA is 1 type of small noncoding RNA that can participate in human physiological and pathological processes. As early as 2005, Golub, TR scholars at MIT, discovered that miRNA profiles could reflect the developmental spectrum and differentiation status of tumors and therefore relied on the expression of miRNA profiles to classify tumors. That stage can illustrate the great significance of miRNAs in tumor diagnosis.^[95]^ And in 2017, scholars from the Third Military Medical University in China discovered that plasma exosomes miR-23b-3p and miR-10b-5p could be used as noninvasive prognostic biomarkers for non-small cell lung cancer, thus clearly pointing out the role of exosomal miRNAs in the diagnosis of respiratory diseases as biomarkers,^[71]^ markers in the diagnosis of respiratory diseases. Similarly, it has also been hypothesized that exosomal microRNAs might act as diagnostic biomarkers for noninvasive screening and monitoring of recurring lung cancer.^[71]^In recent years, it has been shown that micronodular lung cancer can be predicted based on serum extracellular nanovesicle miRNA.^[96]^

## 5. Strengths and limitations

The present research situation and relevant hotspots of the area were analyzed through bibliometric studies on exosomes in respiratory diseases. The research development in this field has a considerable upside, and the exosome’s function in diagnosing, treating, monitoring, and prognosis of respiratory illnesses cannot be taken lightly. In addition, there are some limitations in this study; the bibliometric analysis software has high specifications and standards for data, and only journal papers from the core collection of the Web of Science database were used in this study, which will inevitably lead to the problem of incomplete analysis data.

## 6. Conclusions

The bibliometric analysis combines mathematics, statistics, and bibliography to analyze and interpret the field under study scientifically. Our statistics show that this is the first study of exosomes and respiratory diseases using bibliometrics. In recent years, publications on exosomes in respiratory diseases have shown an increasing trend yearly, representing a significant research value and clinical prospect in this field. China and the United States rank first and second in the number of publications. However, academic study cooperation and communication between the 2 are insufficient, and Chinese-related universities account for a significant proportion of research institutions in this domain. Some heavyweight scholars in this field, such as Clotilde Théry and Takahiro Ochiya, were found to be worthy of our attention and reference. In our study, the core keywords in this field are exosome, lung cancer, expression, activation, mesenchymal stem cell, and mechanism, representing the theme words: lung cancer, and Non-Small Cell Lung Cancer; the most popular fields are Oncology, Cell Biology, Biochemistry & Molecular Biology, and the commonalities are summarized by analyzing many data, we found that the hotspots in this field is focused on lung cancer, MSC-derived exosomes, and exosomal miRNAs. We expect this work will offer valuable references for future studies in this field, contribute to developing exosomes in respiratory diseases, and transform theoretical knowledge into clinical efficacy.

## Author contributions

**Conceptualization:** Jinjie Fu.

**Data curation:** Jinjie Fu, Mengzhen Fan.

**Formal analysis:** Mengzhen Fan, Yang Li.

**Funding acquisition:** Wenjie Song.

**Investigation:** Jinjie Fu, Yang Li.

**Methodology:** Wenjie Song.

**Resources:** Wenjie Song.

**Software:** Jinjie Fu.

**Supervision:** Jinjie Fu, Zheng Hao.

**Validation:** Zheng Hao.

**Visualization:** Jinjie Fu.

**Writing – original draft:** Jinjie Fu.

**Writing – review & editing:** Wenjie Song, Zheng Hao.
